# Fat and inflammation: adipocyte-myeloid cell crosstalk in atherosclerosis

**DOI:** 10.3389/fimmu.2023.1238664

**Published:** 2023-09-15

**Authors:** Aleksandra M. Mazitova, Ana Cristina Márquez-Sánchez, Ekaterina K. Koltsova

**Affiliations:** ^1^ Cedars-Sinai Cancer, Smidt Heart Institute, Cedars-Sinai Medical Center, Los Angeles, CA, United States; ^2^ Department of Medicine, Cedars-Sinai Medical Center, Los Angeles, CA, United States; ^3^ Department of Cardiology, Cedars-Sinai Medical Center, Los Angeles, CA, United States; ^4^ Department of Biomedical Sciences, Cedars-Sinai Medical Center, Los Angeles, CA, United States

**Keywords:** atherosclerosis, perivascular adipose tissue, inflammation, adipocytes, cytokines

## Abstract

Adipose tissue inflammation has been implicated in various chronic inflammatory diseases and cancer. Perivascular adipose tissue (PVAT) surrounds the aorta as an extra layer and was suggested to contribute to atherosclerosis development. PVAT regulates the function of endothelial and vascular smooth muscle cells in the aorta and represent a reservoir for various immune cells which may participate in aortic inflammation. Recent studies demonstrate that adipocytes also express various cytokine receptors and, therefore, may directly respond to inflammatory stimuli. Here we will summarize current knowledge on immune mechanisms regulating adipocyte activation and the crosstalk between myeloid cells and adipocytes in pathogenesis of atherosclerosis.

## 1 Introduction

Atherosclerosis is the most prevalent form of CVD, which accounts for nearly 18 million deaths annually (1). It is a lipid driven, chronic inflammatory disease with progressive growth of atherosclerotic plaques infiltrated with all major immune cell subtypes. Accumulation of lipids particularly within lipid-loaded macrophages (aka “foam” cells) promotes recruitment and activation of inflammatory cells, and production of pro-inflammatory and pro-atherogenic mediators (1–4). Various factors including unhealthy lifestyle(s), suboptimal dietary habits, smoking, stress, and obesity are implicated in the development of atherosclerosis (5).

Obesity is characterized by adipose tissue hypertrophy, expansion of white adipocytes, impaired metabolic homeostasis, and low-grade systemic inflammation that can affect function of multiple organs in the body (6, 7). Adipose tissue also surrounds large blood vessels. Therefore, changes in its environment may dictate inflammatory changes in arteries, thereby also contributing to the pathogenesis of atherosclerosis. While numerous studies showed an important role of adipose tissue in the regulation of metabolism (8–10), the role of PVAT as an “immune organ” impacting inflammation in atherosclerosis is largely unknown.

Myeloid cells, and particularly macrophages, play a dominant role in the pathogenesis of atherosclerosis (11). Furthermore, myeloid cells can be found in adipose tissue, where their numbers and activation status change during obesity progression (12). However, composition of myeloid cell subsets and a crosstalk between adipocytes and immune cells in PVAT is poorly understood. Here we describe current knowledge on how perturbations within adipose tissue can modulate the inflammatory environment and affect myeloid cell accumulation; and discuss potential role of PVAT in the pathogenesis of atherosclerosis.

## 2 Types of adipose tissue

Adipose tissue is composed of adipocytes, and a stromal fraction that includes endothelial and mesenchymal cells, and immune cells (13). Three major types of adipocytes have been described: white, brown, and beige (8, 14). Origin and development of adipocytes has been described in detail elsewhere (15, 16). Brown adipocytes originate from mesodermal progenitors and are typically found in the interscapular area perinatally (17–19). However, during ontogeny their numbers in interscapular area gradually regress; and in adulthood, brown adipose tissue (BAT) is present mostly in neck and supraclavicular regions (20). Brown adipocytes store lipids in small droplets that can be quickly used as energy source, therefore brown adipose tissue plays an important role in thermogenesis (21). Furthermore, brown adipocytes are heavily innervated and vascularized which facilitates substrate and oxygen delivery for efficient thermogenesis (15). White adipose tissue (WAT), which differentiates from mesenchymal stem cells, can be found in visceral and subcutaneous regions (22, 23). Lipids accumulate in all adipocytes primarily in the form of triglycerides; however, brown adipocytes contain more phosphatidyl-choline (PC), phosphatidylethanolamine (PE) and cardiolipin (CL) in comparison to white adipocytes (24). White adipocytes store lipids in the form of single unilocular large lipid droplet (25). The detailed description of lipid composition in various types of adipocytes described elsewhere (24). Upon stimulation of lipolysis, white adipocytes release free fatty acids (FFA) that can be utilized by brown adipocytes as a fuel for heat production via mitochondrial uncoupling or released to the circulation affecting other tissues (26, 27). Beige adipose tissue represents an intermediate state between white and brown; and can change its appearance and function depending on environmental stimuli including temperature, β-adrenergic signaling and nutrients availability (28, 29). In obesity, caloric excess contributes to the transition of beige adipocytes toward white adipocyte phenotype, and their hypertrophy leads to the expansion of visceral and subcutaneous WAT (30, 31).

Apart from the control of energy expenditure and storage, studies for the past decade revealed other important functions of adipose tissue. It becomes increasingly clear that adipose tissue represents an important reservoir of various immune cells (32). Presence of numerous immune cells in adipose tissue positions it as a potential regulator of inflammatory responses. It has been suggested that inflammation in WAT can be initiated by multiple stimuli, including TLR activation via free fatty acid sensing, lipotoxicity, excessive lipid burden-induced ER stress, activation of unfolded protein response, and hypoxia due to inability of blood vessels to grow fast enough to catch up with the rapid expansion of adipose tissue. Overall, the ability of lipid-overloaded WAT to induce and sustain inflammation can lead to enhanced accumulation and activation of immune cells establishing a positive feed-forward loop further fueling the inflammation (32, 33).

**Figure 1:**
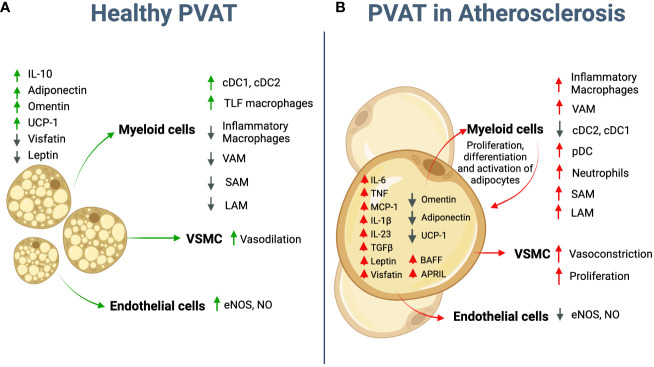
Potential role of adipocytes in atherosclerosis. **(A)** Healthy PVAT is mostly represented by brown adipocytes that secrete anti-inflammatory molecules, such as IL-10, Adiponectin, and Omentin. Crosstalk between adipocyte and immune cells controls adipose tissue homeostasis and vascular tone. **(B)** During atherosclerosis development PVAT adipocytes further differentiate toward “white” pro-inflammatory phenotype. They start to secret multiple pro-inflammatory cytokines such as IL-6, TNF, IL-1β, TGFβ, IL-23, BAFF and APRIL; chemokines, such as MCP-1 and adipokines such as Leptin and Visfatin; and downregulate anti-inflammatory adipokines such as Omentin and Adiponectin. UCP-1 expression essential for energy dissipation and thermogenesis is also downregulated. The crosstalk between adipocytes and infiltrated immune cells further promotes the recruitment of inflammatory macrophages, cDCs, pDCs and neutrophils and overall enhances inflammatory environment in PVAT. Adipocyte-derived Leptin regulates VSMC and EC, and therefore vasoconstriction and endothelial dysfunction. PVAT, Perivascular Adipose Tissue; cDC, conventional dendritic cells; pDC, plasmacytoid dendritic cells; VSMC - vascular smooth muscle cells, EC- Endothelial cells; VAM, vascular associated macrophages; LAM, lipid associated macrophages; SAM, sympathetic neuron-associated macrophages. Created using Biorender.com.

While several common risk factors predispose to both obesity and atherosclerosis, for a long time, these two diseases were viewed as parallel, simultaneously developing, but mechanistically independent. However, alterations in adipose tissue function in atherosclerosis recently started to draw significant attention (34–36). Perivascular adipose tissue (PVAT) is especially interesting due to its proximity to the aortic wall (**Figure 1**). Given the presence of extensive vasa vasorum (multiple vessels and capillary) in PVAT, inflammatory activation of adipocytes and infiltrated immune cells is likely to modulate the inflammation and atherosclerosis either in paracrine manner or by serving as a depot and a source of migrating immune cells (37, 38).

## 3 Perivascular adipose tissue and its role in atherosclerosis

As any other adipose tissue, PVAT is composed of white, brown, and beige adipocytes, stromal and immune cells (39–41). In mice, PVAT in coronary arteries and aortic arch is mostly represented by WAT; while thoracic aorta is surrounded mostly by brown adipose tissue (BAT), and PVAT near abdominal aorta consists of WAT and BAT mixture (40–43). In humans, the exact PVAT topography is less understood and shows heterogenous characteristics between BAT and WAT along the thoracic aorta, while mostly WAT can be found in the abdominal aorta and near mesenteric arteries (44). Recent studies demonstrated hypertrophy of PVAT during atherosclerosis development (45–47), specifically near atherosclerotic-prone sites in the aorta (48). A study by Kim et al. using multimodal nonlinear optical (MNLO) imaging of thoracic PVAT-intact atherosclerotic aorta revealed changes in lipid droplets, collagen, and elastin during plaque growth in atherosclerosis-prone *Apoe^-/-^* mice (45). Hypertrophy of thoracic PVAT adipocytes was detected in *Apoe^-/-^* mice fed with western diet (WD) as compared to C57BL/6 wild type controls; however similar lipid droplet number and size in PVAT adipocytes was observed when mice were fed with chow diet, indicating that increase in adipocyte size and droplet accumulation is happening during progression of atherosclerosis (45). Similar changes in lipid droplet sizes have been observed in humans with atherosclerosis, therefore, adipocyte size and lipid composition of PVAT were proposed as a biomarker to determine the stage of the disease based on so-called fat attenuation index (FAI, i.e. the decrease of lipid content measured by computerized tomography (CT)), that has been validated in patients with coronary artery disease (49). CT studies in human coronary arteries shows a gradient of adipocyte sizes and functional characteristics depending on their proximity to the aortic adventitia. While beige/brown adipocytes are mostly located nearby the adventitia, more white adipocytes appear towards the outer layers of PVAT (49). Nevertheless, the mechanisms underlying this site-specific expansion and transformation remains to be elucidated.

Uncoupling protein-1 (UCP-1), a marker of brown adipocytes, functions to uncouple the mitochondria thereby accelerating fatty acid oxidation, energy expenditure, and thermogenesis. Downregulation of UCP-1 is typically associated with “whitening” of adipose tissue as lipids are no longer actively spent and accumulate. Furthermore, downregulation of UCP-1 reversely correlates with Notch signaling activation (47). The downregulation of UCP-1 was described in patients with coronary artery disease (50) as well as *Apoe^-/-^* mice (45) and was especially prominent in PVAT nearby advanced plaques (45). UCP-1 downregulation, Notch signaling, inhibition of PPARγ-dependent gene expression in adipocytes and TGFβ signaling were suggested to contribute to lipid droplet accumulation and collagen deposition linked to fibrosis near the plaque (45, 47, 49).

Overall, PVAT represents a complex tissue, where type of adipocytes and their inflammatory state depends on the location and changes during atherosclerosis progression. Below we discuss various roles of PVAT in the regulation of vessel tone and inflammatory environment.

### 3.1 Vasoconstriction

Perhaps, the most well-investigated function of PVAT to date is its contribution to blood vessels support and vascular tone (51). The initial indication of PVAT’s involvement in vascular function came from the discovery that PVAT reduced the contractile reactions to noradrenaline in rat aorta (52). Subsequently, it has been established that PVAT is losing its anti-contractile activity in obesity (53). *Ex vivo* studies using aortic rings demonstrate that PVAT from mice fed with normal chow diet promotes vasodilatory effect, while PVAT from High Fat Diet (HFD) fed mice contributed to vasoconstriction (54). The effect on vasoconstriction was partially mediated by stress-response factor ATF3 and was linked to the regulation of potassium channels (54). It was noted that thoracic aortas without PVAT exhibited higher stiffness (loss of blood vessel wall elasticity) supporting vasodilatory role of PVAT. Several mechanisms regulating this phenomenon have been proposed, including secreted by PVAT relaxion factors such as NO (46, 55). In obesity, the relaxing properties of PVAT are abrogated particularly due to reduction in Adiponectin and bioavailability of NO (56).

The effect of PVAT on vasoconstriction is likely mediated via crosstalk between adipocytes and vascular smooth muscle cells (VSMC) (39, 57, 58). Indeed, PVAT was shown to modulate the contractile response of VSMC through a variety of signaling pathways including adipokine signaling, inflammatory signaling, oxidative stress, and metabolic signaling (59). For example, NO released from PVAT contributes to the regulation of vascular tone and blood pressure, controlling VSMC function (59, 60). Adipocyte-derived reactive oxygen species (ROS) can promote VSMC proliferation and migration in extracellular signal-regulated kinase (ERK) signaling-dependent manner thereby contributing to vascular dysfunction and atherosclerosis (61). Recently, PVAT-derived hydrogen peroxide (H_2_O_2_) was also implicated in its anticontractile effect on VSMC as demonstrated *in vitro* on aortic rings co-cultured with PVAT (61). Other metabolites derived from the PVAT, such as hydrogen sulfide (H_2_S) and Angiotensin 1-7 may affect VSMC function by promoting vasodilation (62). For example, H_2_S might promote VSMC relaxation by Ca2^+^-activated K^+^ channels (62).

Furthermore, PVAT was suggested to modulate endothelial cell function. PVAT-derived NO was shown to inhibit the expression of pro-inflammatory cytokines and adhesion molecules (55) and induce the production of anti-inflammatory molecules such as Adiponectin and IL-10 (51), thereby controlling the inflammation and atherosclerosis.

Overall, the PVAT plays an essential role in the regulation of vasodilation relevant to various vascular diseases including atherosclerosis.

### 3.2 Adipokine production

Leptin and Adiponectin are the most abundant adipokines produced by adipocytes in physiological or pathological conditions. Because of its proximity to the vessel wall, PVAT-derived adipokines were suggested to impact the function of various cells in the vessel wall. The expression of adipokines changes in pathophysiological conditions. Hence, the downregulation of adiponectin during obesity is observed, while Leptin is typically elevated (63). Both Adiponectin and Leptin play an important role in the regulation of vascular cells. Adiponectin function was linked to VSMC contractile response via modulation of intracellular calcium levels (64, 65), AMP-activated protein kinase (AMPK) activation, and increase in NO production controlling VSMC relaxation (64, 65). Adiponectin was also shown to regulate endothelial cell function and promote eNOS activity within endothelial cells (66). In accordance, adiponectin-deficient mice spontaneously develop hypertension and chronic endothelial dysfunction (51, 67, 68).

On the other hand, Leptin inhibits the production of adiponectin and regulates VSMC contraction via upregulation of Endothelin 1 (ET-1, a vasoconstrictor and mitogen) (69, 70). It has been also established that Leptin promotes the proliferation (71), migration (72), and neointimal hyperplasia of VSMC via a PI3K-dependent mechanism (73). Leptin was implicated to the regulation of immune cell function (74, 75). Specifically, it was shown to promote TNF, IL-6, IL-12, and ROS production by macrophages in adipose tissue (76). Leptin also plays an important role in T cells. It skews T cell differentiation toward Th17, and ablation of leptin receptor in CD4 T cells limited Th17 cell subset differentiation (77). Moreover, Leptin was shown to inhibit T_regs_ proliferation and induce anergy (78). The deficiency of Leptin (ob/ob mice) or Leptin receptor (db/db mice) limited atherosclerosis development which was accompanied by reduced numbers of IFNγ producing Th1 cells while number of T_regs_ with strong suppressive activity was elevated (74).

Omentin is an adipokine secreted mainly by adipose tissues, including PVAT (79). In patients with CVD Omentin serum levels are reduced (80, 81). *Apoe^-/-^* mice expressing human Omentin transgene in adipocytes and macrophages showed fewer atherosclerotic lesions and reduced macrophage infiltration in the plaque (82), suggesting its anti-inflammatory and anti-atherogenic role. While a few studies suggest that Omentin may regulate VSMC contraction (83) and modulate eNOS expression in the endothelial cells (84), but more mechanistic studies are needed to understand its role in obesity and atherosclerosis.

Visfatin is an adipokine secreted by adipose tissue, and implicated into obesity (85) and atherosclerosis (86). Elevated serum levels of Visfatin have been also reported in patients with carotid atherosclerosis (86, 87). In mouse models, Visfatin has been linked to the foam cell formation via modulation of Scavenger receptors CD36 and SRA expression in macrophages (88) as well as stimulation of VSMC proliferation (89).

### 3.3 Immune cells and cytokine production

While the role of PVAT in the regulation of vascular tone and VSMC draws substantial attention (45, 90), its contribution to the control of immune cell accumulation and activation in atherosclerosis remains less understood. The immune infiltrate to the aortic wall increases both in hypertension and in atherosclerosis. In atherosclerosis, T and B cell were shown to accumulate predominantly in the adventitial margin (35, 91, 92) in close proximity to PVAT. Enhanced immune cell infiltration in PVAT has been documented in *Apoe^-/-^* mice fed with chow diet (91), which was further exacerbated by high-fat diet feeding (47). Immune cell accumulation in PVAT may serve as an important link between vascular and adipose tissue dysfunction and are potent sources of various cytokines affecting aortic and adipose microenvironments (38).

Importantly, adipocytes themselves are able to produce inflammatory cytokines and chemokines, such as TNF, IL-8, MCP-1, IL-6, IL-1β, IL-23, TGF-β, BAFF and APRIL (59, 93–97). The stimulation of pre-adipocyte 3T3-L1 cell line with TNF induces the expression of *p28, Ebi3, p35, p40*, and *p19*, the subunits of IL-23, IL-12 and IL-27 cytokines *in vitro* (96). PVAT collected near the abdominal aorta was shown to produce higher level of pro-inflammatory IL-6 and TNF as compared to thoracic PVAT in rats (98), and heightened MCP-1 expression was found in mouse abdominal PVAT (99). The spectrum and magnitude of produced inflammatory mediators changes during disease development. Hence, PVAT transplanted from C57BL/6 mice to *Apoe^-/-^* mice showed lowered expression of IL-12, IL-6, and MCP-1 which limited macrophage accumulation to the area of transplantation in comparison to *Apoe^-/-^* PVAT in model of hypertension (100).

These observations suggest novel immunoregulatory role of PVAT with yet to be identified mechanisms operating *in vivo* during atherosclerosis progression.

### 3.4 Immune mechanisms of adipocyte activation: specific cytokine signaling in adipocytes

#### 3.4.1 Interleukin-17 signaling and adipocytes

Adipocytes were shown to express various cytokine receptors and consequently, they are responsive to a variety of pro-inflammatory stimuli (101, 102). IL-17 expression is elevated in patients with metabolic syndrome (103, 104). Th17 cells have been linked to the development of atherosclerosis, although their role is not unequivocal (105, 106). IL-17RA is expressed by most cell types, while IL-17RC, a second chain of heterodimeric IL-17R, was recently found to be expressed on adipocytes where it was suggested to control energy expenditure (107). Adipocyte-specific ablation of *Il17rc* (*Il17rc^fl/fl^ AdipoqCre*) resulted in weight gain, increased lipid accumulation in BAT, and glucose intolerance (107). Indeed, when fed with WD, these mice gain weight faster than their Cre negative littermate controls and presented with higher lipid accumulation in BAT, bigger inguinal and epididymal WAT depots and therefore were less tolerant to cold (107). Similar observations were found in mice where IL-17RC signaling was pharmacologically inhibited by antibody (108). Mechanistically it was demonstrated that IL-17A signaling suppresses adipocyte differentiation from 3T3-L1 preadipocytes *in vitro* that correlates with inhibition of transcription factor KLF15 (109). Furthermore, IL-17 signaling may regulate adipocyte metabolism. In obese mice, IL-17 treatment upregulated the expression of multiple metabolic genes, including *Csl1, Atg1, Dio2, Glut4, Nnmt, Hsl, Ucp1, Pgc-1α* and *Acox1* (110). These observations suggest that IL-17 signaling also plays a role in nonimmune tissues and can be an important player in the regulation of adipocyte function in CVD and obesity. Future mechanistic studies focusing on the role of IL-17 signaling in regulation of PVAT in atherosclerosis development would be of a great interest.

#### 3.4.2 Type I and type II interferon signaling in adipocytes

Adipocytes have been reported to produce various interferons, but also express type I and type II IFN receptors and, therefore, are responsive to IFN stimulation (111). Administration of IFNβ to mice with diet-induced obesity restores insulin sensitivity, mitigates the expansion of adipose tissue and weight gain, and increases thermogenesis (112). Adipocytes stimulated with type I IFN (IFNβ) demonstrate transcriptional signature very similar to IFN/LPS treated myeloid cells (111). Furthermore, IFNα/IFNαR signaling was implicated in the regulation of glycolysis in adipocytes (111). While whole body IFNαR knockout developed obesity similarly to WT controls, it presented with different distribution of WAT with hypertrophy, enhanced death of adipocytes in epididymal-eWAT and reduction of inguinal and perirenal WAT. Ablation of IFNαR reduced the accumulation of immune infiltrate in eWAT (111). Furthermore, adipocyte-specific ablation of IFNαR in *Ifnar^fl/fl^ AdipoqCre* mice fed with HFD revealed a significant reduction of inflammatory cytokine production from adipocytes (111).

Adipocytes were also shown to express type II IFN receptor IFNγR (113–115). The IFNγ/IFNγR signaling pathway has been shown to play a role in the regulation of adipose tissue inflammation (116). *In vitro* studies with 3T3-L1 cells suggest that IFNγR signaling might be involved in the regulation of lipid metabolism in adipocytes as IFNγR stimulation downregulated lipoprotein lipase and fatty acid synthase (117), and also exerts a downstream activation of the STAT1/3 pathway resulting in inhibition of PPARγ expression (116). Overall, these observations suggest that IFNαR and IFNγR signaling are fully functional in adipocytes and regulate their inflammatory activation.

#### 3.4.3 Interleukin-6/IL-12 superfamily signaling in adipocytes

The IL-6/IL-12 superfamily includes IL-6, IL-12, IL-23, IL-27 and IL-35 cytokines. These cytokines connect innate and adaptive immune responses and can exert pro-inflammatory and anti-inflammatory effects in context dependent manner (118–121). The IL-6/IL-12 superfamily transduces their signals through receptor complexes represented by heterodimers (121) with one of the subunits, for example Gp130 expressed on all cell types throughout the body, and another one with more cell type specific expression, such as membrane-bound IL-6Rα that can be found on hepatocytes, epithelial cells, leukocytes and adipocytes (122, 123). Recent studies began to illuminate the role of these cytokines in the regulation of adipocyte function.

##### 3.4.3.1 Interleukin-6 signaling in adipocytes

In adipocytes, IL-6 signaling can be mediated by classical trans-membrane IL-6R (formed by the heterodimer IL-6Ra and gp130) (124, 125), or by trans-signaling mediated by soluble IL-6 Receptor (sIL-6Ra) which binds to IL-6 and surface-expressed gp130 (123). IL-6 signaling promotes Leptin secretion and lipolysis in BAT adipocytes, as well as induce energy expenditure (126). Moreover, chronic activation of IL-6/IL-6R signaling in adipose tissue was linked to the development of obesity-related metabolic disorders, such as insulin resistance and type 2 diabetes (123, 127). IL-6 regulates energy expenditure in obese individuals and may also act as a first homeostatic response to low-grade inflammation related to obesity. In healthy humans, IL-6 was linked to high insulin sensitivity and fatty acid oxidation (125). On another hand, Adiponectin production by adipocytes was suppressed by IL-6 (128), implying that IL-6 signaling in adipocytes play an important role in the regulation of adipokine production, that in turn may control the inflammation and vascular dysfunction. IL-6 is also known to promote recruitment of various immune cells via control of pro-inflammatory chemokines production by myeloid cells (95) and, possibly, adipocytes.

However, detailed mechanisms of IL-6 signaling in PVAT adipocytes and their specific role in atherosclerosis remain to be further investigated.

##### 3.4.3.2 Interleukin-27 signaling in adipocytes

IL-27R signaling has been recently implicated in the regulation of adipocyte function, and individuals with obesity show a significant decrease in serum IL-27 (129). IL-27R deficient mice were found to be susceptible to HFD-induced obesity and develop insulin resistance, glucose intolerance and steatohepatitis. Both leptin and adiponectin levels were elevated in the circulation of IL-27 deficient mice. Adipocyte-specific ablation of IL-27R using *Il27ra^fl/fl^ AdipoqCre* or *Il27ra^fl/fl^UCP1-Cre-ERT* mice resulted in increased HFD-induced obesity and metabolic syndrome (129), although IL-27R ablation in brown adipocytes showed milder effect. Furthermore, these mice show significantly diminished thermogenesis, reduction of UCP-1 expression and less multiocular lipid droplets in adipocytes (129). Conversely, no changes were found in body weight gain in mice lacking IL-27Ra in immune cells (129). The administration of recombinant IL-27 to wild-type mice reduced body weight, adipose deposition, and improve insulin resistance, while the protective effect was ameliorated both in *Il27ra^fl/fl^ AdipoqCre* and *Il27ra^fl/fl^ UCP-Cre-ERT2* mice (129). Mechanistically, stimulation of IL27R-sufficient primary beige adipocytes *in vitro* with rIL-27 induced UCP1, PPARα, and PCG1-α expression, a main regulators of energy metabolism; in p38 MAPK and ATF2-dependent manner (129). These data demonstrate that IL-27R signaling can directly modulate the metabolism of adipocytes. Future studies will be needed to evaluate how IL-27R signaling in adipocytes may regulate the inflammatory environment in PVAT in atherosclerosis.

**Figure 2:**
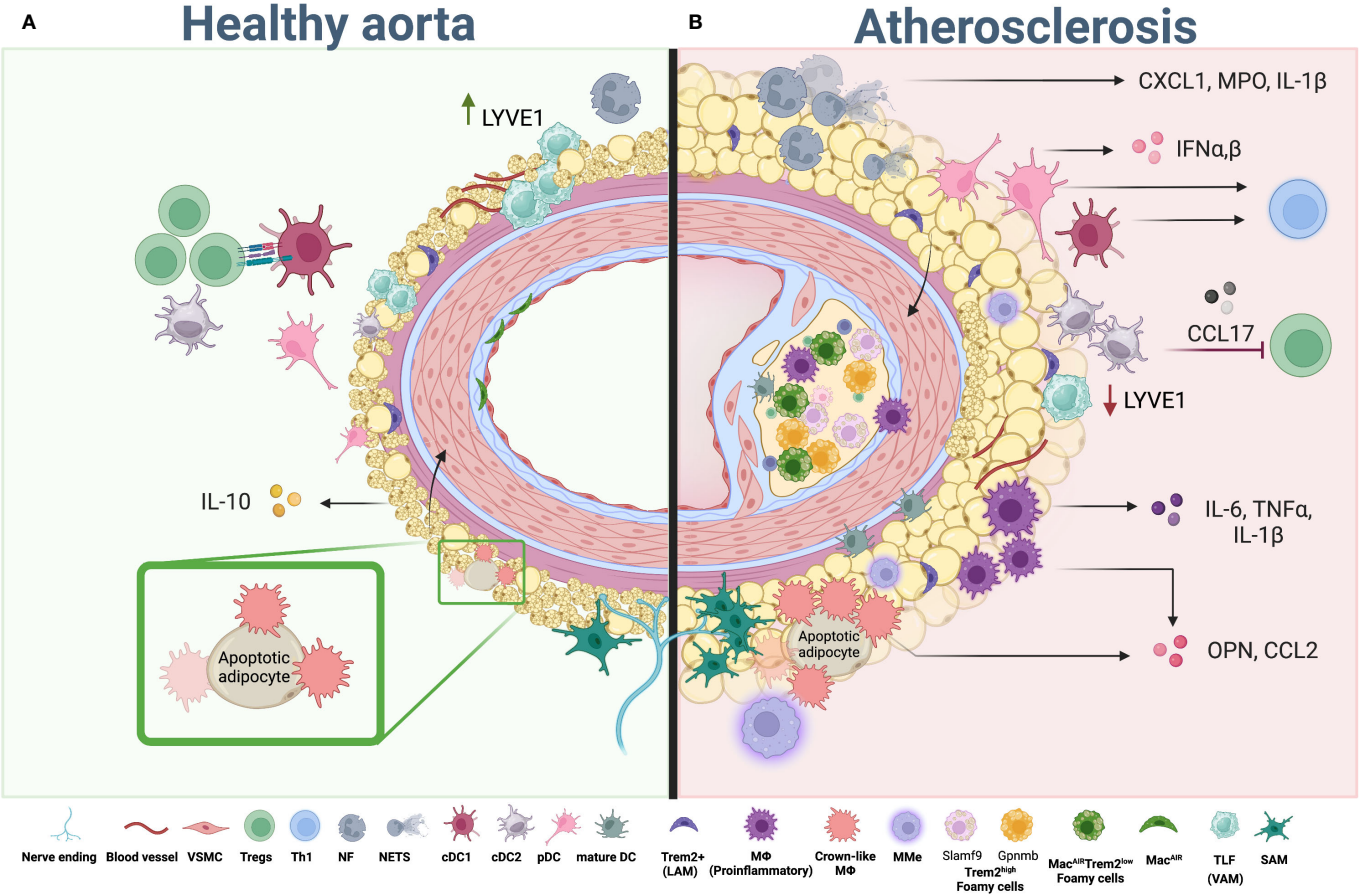
Myeloid cells in aortic PVAT and atherosclerosis. **(A)** Healthy PVAT is composed of brown and white adipocytes and infiltrated with immune cells. Composition of myeloid cells in healthy PVAT is represented by VAM, TLF, SAM, “Crown-like” macrophages, cDCs (cDC1 and cDC2) and a small number of neutrophils. While macrophages maintain tissue homeostasis, DC can activate anti-atherogenic T_reg_ cells, helping to maintain immune tolerance in the tissue. **(B)** During atherosclerosis development whitening of adipocytes and expansion of adipose tissue occurs alone with changes in the composition of myeloid cells. Pro-inflammatory, lipid associate (Trem2^high^ and Trem2^low^) and metabolically activated macrophages (MMe) start to accumulate in adipose tissue and aorta, releasing pro-inflammatory chemokines and cytokines including IL-1β, TNF, IL-6, CCL2 and OPN, which in turn activate adipocytes in PVAT and provide a positive feedback loop to adipocyte whitening, inflammatory activation and subsequent pathogenic changes within PVAT. Neutrophils in adipose tissue accumulate in high numbers resulting in enhanced release of NETs and pro-inflammatory IL-1β and CXCL1. Numbers of dendritic cells, including cDC, mDC and pDC, are also increased. While cDC exert their function via antigen presentation to control T cell activation and differentiation, pDC regulate the inflammatory environment via type I IFNs production. Th1 and other T helper subsets; T_regs_, regulatory T Cell; PVAT, perivascular adipose tissue; VSMC, vascular smooth muscle cells, cDC; conventional dendritic cells; pDC, plasmacytoid dendritic cells; mature DC, mature dendritic cells; NF, neutrophil; NETs, neutrophil extracellular trap; TLF, Timd4^+^Lyve1^+^Folr2^+^ cells (vascular associated macrophages); LAM, lipid associated macrophages; SAM, sympathetic neurons associated macrophages; MMe, metabolically activated macrophages; Mac^AIR^, Aortic intima resident macrophages. Created using Biorender.com.

Taken together, while multiple evidence demonstrated an important role of PVAT in blood vessels support and control of vascular tone, emerging data suggest that it could also play a key immunoregulatory role (**Figure 2**). Recently described expression of cytokine receptors in adipocytes suggest novel roles of cytokines in the control of this tissue type, which in turn may regulate immune cell accumulation. Further studies addressing how cytokine may regulate adipose tissue including PVAT will help to shed light on novel mechanisms regulating this tissue and its impact on immune cell accumulation and activation in atherosclerosis development.

## 4 Myeloid cells in adipose tissue and atherosclerosis

### 4.1 Macrophages

Macrophages are innate myeloid cells, which play key roles in the maintenance of tissue homeostasis as well as in inflammatory responses (130). Tissue resident macrophages, originating from the yolk sac during embryogenesis are more specialized in initial maintenance of homeostasis and play sentinel functions (131, 132). In response to the tissue injury-initiated inflammation, monocytes are recruited to the injured area and give rise to monocyte-derived macrophages (133). The function of macrophages can be site- and tissue- specific (134). The wide spectrum of macrophage activation can be captured in the tissue, and state of macrophage activity as well as spectrum of produced molecules is determined by environmental cues (130).

Macrophages also are the most abundant population of immune cells in adipose tissue and are key regulators of adipose tissue homeostasis (12). In lean state, the macrophage population in adipose tissue is represented mostly by anti-inflammatory/tissue-repair/alternatively activated subset (135). Obesity facilitates the accumulation of pro-inflammatory macrophages (135). Several subsets of adipose tissue macrophages have been described. All these subsets are established based on their localization or transcriptional signatures (136). In adipose tissue, macrophages closely interact with adipocytes. Representation of different macrophage subsets in PVAT is poorly characterized; and the role of the crosstalk between these two cell types in aortic inflammation and atherosclerosis is not well understood. Since adipocytes in PVAT can produce inflammatory cytokines and chemokines (59, 94, 98, 99), it is plausible that their inflammatory activation may be implicated to the recruitment and activation of macrophages, which due to the physical proximity to the aorta will likely impact aortic wall inflammation and thus, atherosclerosis.

In atherosclerotic aortas macrophages represent the most abundant immune cell population known to regulate the disease development at different stages (11, 137). Recent studies using single-cell RNA sequencing (scRNAseq) of isolated immune cells from the aorta or adipose tissue revealed a wide spectrum and continuum of macrophage activation, which significantly enriched our understanding of population-specific markers. Integrated analysis of twelve different scRNA sequencing datasets of macrophages from healthy and atherosclerotic mouse aortas revealed four main macrophage subsets in mice: tissue-resident (*Lyve1^+^, Timd4^+^, Cd209f^+^*), inflammatory (*Ccr2^+^*, MHCII genes, *Il1b^+^, Cd74^+^*), which also include aortic plaque inflammatory (*Cxcl2^high^, Cd14 ^high^*), foamy (*Trem2^+^*) and aortic intimal resident macrophages (Mac^AIR^) (137–139). Human arteries, however, appear to contain three major macrophage populations: Inflammatory-Mφ (*CD74, HLA-DRB1*), Foamy-Mφ (*APOC1, APOE, FABP5, FABP4*) and LYVE1-Mφ (*LYVE1, LGMN, MARCO*) (138) (**Figure 2**).

#### 4.1.1 Functional subtypes of macrophages

Diversity of macrophages in adipose tissue has been extensively studied; and markers and functional characteristics of adipose tissue macrophages have been well-described (136).

##### 4.1.1.1 Crown-like macrophages

One important function of macrophages in adipose tissue is scavenging of debris and dead cells (140). Macrophages that cluster around dying adipocytes are known as “crown-like” macrophages, and their numbers are increasing in obesity (141). Damaged adipocytes release lipids and damage associated molecular patterns (DAMPs) that in turn activate macrophages infiltrating adipose tissue. “Crown-like” macrophages produce CCL2 (142), and Osteopontin, a pro-atherogenic mediator (119), which has been also implicated to the control of adipocyte progenitors accumulation and differentiation. This interplay between adipocytes and macrophages highlights the complex interactions within adipose tissue (143) and dysregulation of this process may have implications for metabolic and cardiovascular health.

##### 4.1.1.2 Metabolically activated macrophages

High level of glucose and fatty acids in obesity promotes metabolically activated transcriptional profile in macrophages (144). Similarly, to “crown-like” macrophages, these cells are located nearby dying adipocytes and engulf them. Metabolically activated macrophages (MMe) produce lipases, essential for the fat digestion, and internalize lipids released by dying adipocytes, which in turn promote their activation in PPARγ, p62 and NOX2-dependent manner (144). Furthermore, MMe was shown to potentiate inflammation and express high level of pro-inflammatory genes (*Il1b, Tn, Il6*) as well as genes involved in lipid metabolism (*Plin2, Cd36, Abca1*) (145) and, thus, their presence correlated with augmented adipose tissue inflammation (145). These cells create lysosomal synapses with dying or dead adipocytes in order to prevent lipotoxicity caused by necrotic adipocytes (143). PPARγ plays an important role in regulation of cell metabolism and function. Constitutive acetylation of PPARγ in macrophages impedes their ability to skew toward anti-inflammatory state, and mice with constitutive acetylation-mimetic form of PPARγ in macrophages shows heightened macrophage infiltration to adipose tissue and activation toward pro-inflammatory state (146). In atherosclerosis, multiple macrophage populations detected in the plaque and vessel wall have at least some characteristics of MMe and can be accounted also as inflammatory macrophages. Recent scRNA seq analyses identified them as *Ccr2+*, *Cd74*+ and *H2-Eb1*+. They can also express *Nlrp3* and *Il1b*. Another small cluster of inflammatory macrophages are Interferon-inducible macrophages that are characterized high expression of *Ifit3, Irf7*, and *Isg15* genes (147). During atherosclerosis progression pro-inflammatory macrophages accumulate in the aortic wall and atherosclerotic plaque and secrete pro-inflammatory, pro-atherogenic cytokines including TNF, IL-1β, IL-6, IL-12 and others (137).

##### 4.1.1.3 Lipid-associated macrophages

Another distinct population of macrophages found in adipose tissue are lipid-associated macrophages (LAM) (148). Gene expression signature of LAM macrophages resembles that of foam cells in atherosclerotic plaque and is represented by the expression of characteristic genes such as *Lgals3, Ctsb, Itgax*, and especially *Trem2* (149). These cells surround adipocytes and are characterized by the expression of the lipid-detecting receptor - triggering receptor expressed on myeloid cells 2 (Trem2), and genes encoding proteins related to lipid uptake, lipid catabolism and phagocytosis. The number of these cells is increased in adipose tissue during obesity (148). Genetic ablation of *Trem2* results in limited recruitment of macrophages to the expanding adipose tissue and, thus, contributing to massive adipocyte hypertrophy, systemic hypercholesterolemia, inflammation, and glucose intolerance (139, 148). Therefore, Trem2^+^ LAM macrophages are essential for the maintenance of adipose tissue homeostasis and control of metabolic diseases including atherosclerosis (139, 148). Another subset of tissue-resident macrophages was found in the inguinal white adipose tissue (iWAT) and eWAT. These cells are characterized by TIM4 expression and production of PDGF-family growth factors and facilitate lipid storage in adipose tissue in response to dietary changes (150).

In atherosclerosis Trem2^+^ LAM macrophages had been identified in mouse and human arteries (139). They are located in aortic plaque and are characterized by *Igtax* (CD11c), *Trem2*, *Cd9* and *Spp1* expression, at least in mice (138, 151). Trem2 is implicated in lipid influx and foam cell formation in atherosclerosis (152), and Trem2^+^ LAM upon lipid uptake become foam cells (138). *Trem2* genetic ablation significantly reduces atherosclerotic plaque and lipid accumulation in the plaque (152). In mouse aorta Trem2^+^ LAM can be further divided onto two subpopulations: Trem2^hi^ *Slamf9* and Trem2^hi^ *Gpnmb*. Trem2^hi^ *Slamf9* cluster is enriched for *Cd72, Ch25h*, and inflammatory markers (*Tnf, Il1b*), while Trem2^hi^ *Gpnmb* expresses *Gpnmb, Syngr1* and *Fabp5* (138). In humans, a population of LAM macrophages expressing PLIN^hi^/TREM1^hi^ had been recently reported (153). The presence of PLIN^hi^/TREM1^hi^ macrophages was higher in patients who experienced stroke or transient ischemic attack (153). RNA-velocity trajectory analysis suggests that TREM2^hi^ macrophages differentiate toward inflammatory PLIN^hi^/TREM1^hi^ LAMs (153), and oxLDL uptake further upregulates *PLIN2* expression. PLIN^hi^/TREM1^hi^ LAMs are characterized by the enrichments of genes associated with apoptosis and inflammation such as *G0S2, BTG1, BCL2A1, IER3, BNIP3L* (153). OxLDL was recently shown to regulate metabolism of aortic macrophages, modulating fatty acids trafficking to mitochondria and suppressing OXPHOS in CD36-dependent manner (154), and therefore can be further implicated in regulation of *in situ* macrophages phenotypic plasticity.

Another key subset of macrophages in the aorta was shown to take up lipids becoming foam cells. They are found in intimal layer and accumulate with atherosclerosis progression (155). Recent studies identified them as Mac^AIR^ macrophages (137). Mac^AIR^ are characterized by *Acp5, Cd74, Mmp12* and *Gnt2* gene expression (153). They also express *Vcam1, Fcgr4*, but have low level of *Trem 2* and *Spp1*. Recent evidence suggests that they maybe in direct contact with vessel lumen (138). While Mac^Air^ macrophages are differentiate come from monocytes, they are able to maintain independently of circulating cells via local proliferation (137). Mac^AIR^, Trem2^hi^ *Gpnmb* and Trem2^hi^ *Slamf9* subset have overlapping functions including cellular response to lipids. However, each population may have also unique functions as well. For example, Mac^AIR^ macrophages express more genes associated with antigen presentation, Trem2^hi^ *Gpnmb* macrophages are involved in osteoclast differentiation, while Trem2^hi^ *Slamf9* cells negatively regulate macrophage colony stimulating factor (M-CSF) pathway (153).

##### 4.1.1.4 Vasculature associated macrophages

Vasculature associated macrophages (VAMs) are resident macrophages located near blood vessels in adipose tissue (156). The inflammatory VAMs are characterized by expression of LYVE1, which binds hyaluronan on VSMC, and bring these cells together to allow matrix and collagen remodeling in MMP9-dependent manner (157). VAM macrophages can upregulate LYVE1 in response to local hypoxia, and loss of LYVE1 was associated with reduced presence of blood vessels in adipose tissue indicating the role of this subtype in regulation of adipose tissue vascularization (143, 158–160). VAMs numbers are constantly and dynamically changing. They increase in response to high-fat diet feeding, while decrease during fasting or treatment with β3-adrenergic agonists which induce lipolysis (160). Acute inflammation induced by Lipopolysaccharide (LPS) or *Salmonella enterica* markedly reduces the number of VAMs (160). Adipose tissue macrophages express high level of Neuropilin-1 (*Nrp1*) and Nrp1 macrophage-specific ablation results in compromised glucose tolerance, weight gain and reduced efficiency of fatty acid catabolism (159).

In atherosclerosis adventitia’s tissue-resident macrophages have been identified. They are CSF1 dependent and are characterized by high level of *Lyve1, Tim4* and *Folr2* expression and, therefore, in resent publications, have been named TLF. This population can be further subdivided on TLF-*Cd209^hi^* and TLF-*Cd209^low^* (161). Although, the unique function of these subsets remains to be established, early studies identified these cells as tissue-repair macrophages known to produce Arginase-1 and chitinase-like protein 3 (Chil3). They express CD209, CD163 and CD206 surface markers and metabolically rely mostly on OXPHOS (4, 162). This subset was originally suggested to play a protective role in vessel homeostasis and implicated in plaque regression (163). Numbers of tissue repair macrophages are typically decrease during the progression of atherosclerosis, while plaque regression is associated with heightened presence of this anti-inflammatory tissue-repair cell type (163). In humans, the presence of tissue repair macrophages was associated with calcification in atherosclerotic plaque, and heightened intima and media thickness, a signature of plaque stability (151).

##### 4.1.1.5 Sympathetic neuron-associated macrophages (SAMs)

Nervous system plays an important role in the regulation of adipose tissue homeostasis and energy storage (164). Changes in temperature or availability of nutrients drive catecholamine production from adrenal glands, which have a significant effect on the regulation of central nervous system-adipose tissue crosstalk, promoting lipolysis and energy expenditure via β-adrenergic receptor activation by noradrenaline (165). Recently, the communication between macrophages and neurons has been demonstrated. Thus, macrophages interacting with neurons have been named “sympathetic neuron- associated macrophages” (SAMs). These macrophages are located near sympathetic neurons and can import and degrade norepinephrine (NE) leading to decreased NE levels in the tissue and reduction in lipolysis in WAT which leads to increased weight gain (165).

Number of SAMs during obesity is typically increased, and their transcriptional profile shows heightened expression of genes associated with neuronal development and synaptic signaling (165). These macrophages express a noradrenaline transporter SLC6A6 and monoamine oxidase (MAO). Genetic ablation of *Slc6a6* enhanced thermogenesis and adipocyte browning (165). Methyl-CpG-binding protein 2 (MeCP2) is a transcriptional regulator that plays a critical role in development and function of neurons, but also other immune cells including macrophages. *Mecp2* expression in macrophages can be influenced by inflammatory signals, such as LPS and cytokines. It was shown that *Mecp2* ablation in brown adipose tissue macrophages impaired sympathetic innervation and therefore promoted spontaneous obesity via altered adipose tissue thermogenesis (166).

Neuro-immune interaction has been recently implicated in enhanced immune cell activation and cytokine production in atherosclerosis (167). Activation of sympathetic nervous system was linked to atherosclerosis development, particularly via control of hematopoiesis (168). Catecholamines produced by leukocytes and sympathetic nerve in bone marrow promote expansion of GMPs and myeloid cell output required for enhanced atherosclerosis development in diabetic WD-fed *Apoe^-/-^* mice (169). The expansion of neurons was also detected during the progression of atherosclerosis in WD-fed *Apoe^-/-^* mice. The crosstalk between neurons and macrophages in atherosclerosis may be especially prominent in PVAT which is heavily innervated.

While certain mechanisms discussed herein may also be directly applicable to PVAT, future studies will be needed to establish the composition and function of macrophages in PVAT and determine the role of adipocyte-macrophage cross-talk on inflammatory environment in the aorta in atherosclerosis.

### 4.2 Neutrophils

Neutrophils are myeloid cells which are first responders at to the sites of inflammation (170). Their recruitment is mediated by chemokines including CXCL1, CXCL8, complement fragments and bacterial peptides (171). Activated neutrophils produce cytokines, elastase (NE), defensins, myeloperoxidase (MPO) as well as extracellular traps (NETs), which are intricate web-like structures entrapping pathogens and extracellular entities (171, 172). LPS, cytokines, and cholesterol crystals mediates neutrophils activation and NETs release in atherosclerosis (173). In advanced stages of atherosclerosis, neutrophils can contribute to plaque destabilization and rupture (174). Their activation and release of proteases, such as matrix metalloproteinases (MMPs), can weaken the fibrous cap of the plaque, making it prone to rupture (174). Plaque rupture can trigger the formation of blood clots, leading to acute cardiovascular events.

Neutrophils were detected in adipose tissue, although they represent a rather minor population in lean eWAT (175). During obesity neutrophil numbers are rapidly increased (up to 20 times) both in eWAT (175) and in PVAT (176). Inflamed adipocytes producing IL-8 and other chemokines were suggested to mediate neutrophil recruitment into adipose tissue (200). Accumulated in adipose tissue Neutrophils were shown to produce CCL2 and TNF, that in turn facilitate the recruitment of monocytes (176). Activation of Neutrophils by fatty acids released from adipocyte results in IL-1β (177) and ROS production governs the recruitment of other immune cells (**Figure 2**).

### 4.3 Dendritic cells

Dendritic cells are professional antigen-presenting cells that are crucial for T cell activation (178, 179). Several subsets of DC have been identified, including conventional (cDC) and plasmacytoid pDC (180). cDC can be further divided into two main subsets based on their phenotype and function: cDC1 and cDC2 (181). In addition to standard surface markers, recent scRNA-sequencing analyses proposed additional transcriptional signatures to identify cDC subsets in aortas. Mouse cDC1 are characterized by *Xcr1* and *Clec9a* expression, while human cDC1 express *CLEC9A, IRF8* and *IDO1.* Mouse cDC2/monocyte-derived DC are characterized by *Cd209a, Clec10a, Ifitm1* and *Napsa* gene expression, while human cDC2 express *CLEC10A, FCER1A* and *CD1C.* Mouse aortic mature *Fscn1^+^ Ccr7^+^* DC also express *Il4i1^+^, Cd274^+^, Tnfrsf4^+^, Ccl22^+^, Cd40^+^ and CD86* genes (138).

In adipose tissue, cDC1 promote differentiation of regulatory T cells, which help to suppress inflammation and prevent the development of obesity and metabolic dysfunction (182). The ablation of CD11c^+^CD8^+^ cDC1 in *Batf3^-/-^* mice led to weight gain, while the expansion of cDC1 caused the weight loss and increase in numbers of T_regs_ and iNKT (invariant Natural Killer) (182). At the early stage of atherogenesis, DC have been identified in the subintimal space, where they were shown to uptake lipids (180), however recent scRNA seq studies suggest that these CD11c^+^ cells are actually similar to Mac^Air^ macrophages. During atherosclerosis progression DC accumulate in the aorta, particularly in adventitia where they present antigens to CD4 T cells and activate them directly in the aortic wall (183). CCL17-expressing DC were suggested to restrain T_reg_ responses thereby contributing to atherosclerosis development (184). Clec4a4 or DCIR2 (Dendritic cell immunoreceptor 2) is a C-type lectin receptor, which is expressed by CD8α^-^ cDC. WD-fed *Ldlr^-/-^* mice lacking *Clec4a4* developed smaller plaques with only limited necrosis indicating pro-atherogenic role of this DC subset. *Ldlr^-/-^Clec4a4^-/-^* mice were characterized by lower plasma cholesterol and triglyceride levels, as well as fewer monocytes and neutrophils in circulation suggesting that Clec4a4^+^ DC may regulate mobilization of myeloid progenitors from the bone marrow under hypercholesterolemic conditions (185). cDC2 (CD11c^+^CD11b^+^CD8^-^) was also shown to play an important role in the regulation of T cell immunity (178). Activation of PPARγ in cDC2 suppresses the onset of local inflammatory responses in adipose tissue during inflammation by promoting the differentiation of T_regs._ During atherosclerosis development cDC2 were reduced in aortas of WD-fed *Apoe^-/-^* mice (186).

High-fat diet feeding causes the expansion of pDC in visceral adipose tissue. They produce significant amounts of IFN, which suppresses the accumulation of PPARγ^+^ T_regs_ by affecting their proliferation and survival (187). Pharmacological or genetic depletion of pDC by anti-PDCA-1 antibody or in *BDCA2^DTR^* mice treated with diphtheria toxin, lowered body weight and blood glucose level and contributed to the expansion of T_reg_ cells (187). pDC have been found in atherosclerotic lesions, where they may play a dichotomous role during development and progression of the disease (155, 188). At early stages, pDC may contribute to the initiation of the disease by rapidly secreting type I interferons (IFNs), which promote foam cell formation (189). pDC also release pro-inflammatory cytokines, such as TNF, and enhance the recruitment and activation of T cells and monocytes (190). In human atherosclerosis, IFNα secretion correlated with plaque instability (191). IFNα stimulation promotes production of IFNγ and TRAIL by CD4^+^ T cells which in turn may contribute to vascular smooth muscle cell death in antigen-independent manner (191). However, at advanced stages of atherosclerosis, pDC may be atheroprotective and limit the disease progression by dampening proliferation and activation of T cells (192).

Little is known about the roles of DC specifically in PVAT, although they were found at adventitia-PVAT border (193) (**Figure 2**). Presence, activation status and functions of various DC subsets in PVAT has not been yet defined. The inflammatory changes in PVAT may facilitate DC accumulation and activation acting *via* secretion of adipokines and cytokines such as Leptin, Resistin, and TNF (194, 195). Growing and inflamed PVAT can produce chemokines, such as CCL2 and CXCL8, which attract DC to the site of inflammation.

## 5 Concluding remarks

Perivascular adipose tissue is a dynamic and metabolically active tissue that interacts with vascular wall and immune system. The role of PVAT in atherosclerosis is now gaining attention, not only as a regulator of vasoconstriction, but as a source of paracrine molecules. Furthermore, PVAT as any other adipose tissue also serves as a reservoir for various immune cells, including myeloid cells. These immune cells contribute to the chronic low-grade inflammation in PVAT and may regulate the progression of atherosclerosis. In the past two decades, multiple molecules secreted within PVAT and regulating aortic tissue in a paracrine manner has been identified, but the cellular source and mechanisms of action on various cells within the aortic wall remains incompletely understood. Increased cytokine production and immune infiltration into PVAT has been reported both in obesity and hypertension, promoting the pro-inflammatory crosstalk between immune cells and adipocytes, however, the specific changes in immune cell composition in PVAT during atherosclerosis development remains to be determined.

It is likely that under inflammatory conditions during the development of atherosclerosis, interaction between adipocytes and infiltrating myeloid cells will generate a positive feed-forward loop potentially facilitating the recruitment of pro-inflammatory myeloid cells and, thus, further fueling the inflammation in the aortic wall. The pro-inflammatory signaling might induce adipocyte differentiation from BAT-like to WAT-like, and therefore stimulates the production of various cytokines and adipokines. Defining new mechanisms regulating the crosstalk between adipocytes, PVAT infiltrating myeloid cells and aortic wall/tissue may help to develop targeted therapies or preventive approaches in CVD. By studying the heterogeneity of myeloid cells in PVAT, we can gain insights into the complex connections between inflammation in adipose tissue, immune responses, and atherosclerosis. Nevertheless, the causality and the level of participation of PVAT in the development, stability, and rupture of the atherosclerotic plaques need to be further elucidated.

## Author contributions

AM and AS prepared the Figures, AM, AS and EK wrote the manuscript. All authors contributed to the article and approved the submitted version.
